# Reduced T wave alternans in heart failure responders to cardiac resynchronization therapy: Evidence of electrical remodeling

**DOI:** 10.1371/journal.pone.0199637

**Published:** 2018-06-28

**Authors:** Sachin Nayyar, Adrian Suszko, Andreu Porta-Sanchez, Rupin Dalvi, Vijay S. Chauhan

**Affiliations:** Peter Munk Cardiac Center, Division of Cardiology, University Health Network, Toronto, ON, Canada; University of Minnesota, UNITED STATES

## Abstract

**Background:**

T-wave alternans (TWA), a marker of electrical instability, can be modulated by cardiac resynchronization therapy (CRT). The relationship between TWA and heart failure response to CRT has not been clearly defined.

**Methods and results:**

In 40-patients (age 65±11 years, left ventricular ejection-fraction [LVEF] 23±7%), TWA was evaluated prospectively at median of 2 months (baseline) and 8 months (follow-up) post-CRT implant. TWA-magnitude (V_alt_ >0μV, k≥3), its duration (d), and burden (V_alt_ ·d) were quantified in moving 128-beat segments during incremental atrial (AAI, native-TWA) and atrio-biventricular (DDD-CRT) pacing. The immediate and long-term effect of CRT on TWA was examined. Clinical response to CRT was defined as an increase in LVEF of ≥5%. Native-TWA was clinically significant (V_alt_ ≥1.9μV, k≥3) in 68% of subjects at baseline. Compared to native-TWA at baseline, DDD-CRT pacing at baseline and follow-up reduced the number of positive TWA segments, peak-magnitude, longest-duration and peak-burden of TWA (44±5 to 33±5 to 28±4%, p = 0.02 and 0.002; 5.9±0.8 to 4.1±0.7 to 3.8±0.7μV, p = 0.01 and 0.01; 97±9 to 76±8 to 67±8sec, p = 0.004 and <0.001; and 334±65 to 178±58 to 146±54μV.sec, p = 0.01 and 0.004). In addition, the number of positive segments and longest-duration of native-TWA diminished during follow-up (44±5 to 35±6%, p = 0.044; and 97±9 to 81±9sec, p = 0.02). Clinical response to CRT was observed in 71% of patients; the reduction in DDD-CRT paced TWA both at baseline and follow-up was present only in responders (interaction p-values <0.1).

**Conclusion:**

Long-term CRT reduces the prevalence and magnitude of TWA. This CRT induced beneficial electrical remodeling is a marker of clinical response after CRT.

## Introduction

Mechanical coordination of left ventricle (LV) with long-term cardiac resynchronization therapy (CRT) has been shown to reverse LV structural and molecular remodeling in cardiomyopathy patients with wide left bundle branch block (LBBB) and LV dysfunction. In particular, sarcomeric proteins, sarcoplasmic reticulum calcium ATPase 2α (SERCA) and ryanodine receptor (RyR) levels are restored, resulting in augmentation of excitation-contraction coupling and intracellular calcium cycling [1,2]. In addition, restoration of electric LV conduction direction may reverse chronic LBBB-related action potential duration (APD) heterogeneity arising from long-term cardiac memory [3,4].

The effect of CRT on cardiac repolarization heterogeneity, a ventricular arrhythmia (VA) risk marker remains under studied. There are no longitudinal data that have tracked changes in ventricular repolarization with clinical response after long-term CRT. Microvolt T-wave alternans (TWA) arises from abnormal intracellular calcium cycling, due in part to reduced SERCA in cardiomyopathy, and is an indicator of ventricular repolarization instability [5]. Accordingly, we tested the hypothesis that long-term CRT will attenuate TWA in cardiomyopathy patients. Furthermore, these changes in TWA will be associated with mechanical LV improvement and a reduction in VA burden.

## Methods

### Study population

Forty patients with ischemic or non-ischemic dilated cardiomyopathy undergoing CRT with defibrillator (CRT-D) implantation as per heart-failure management and sudden cardiac death prevention guidelines were prospectively enrolled. All patients had a baseline echocardiographic LV ejection fraction (EF) ≤ 35%, native QRS duration >130ms, New York Heart Association (NYHA) functional class ≥ 2 and had received guideline recommended optimal medical therapy for at least 3-months prior to consideration of CRT. Patients with heart-failure undergoing an upgrade of implantable cardioverter defibrillator (ICD) to CRT-D or those with a history of spontaneous VA were also included. Patients with an AV-block indication for pacing, recent acute coronary event in the last 1-month, and decompensated heart-failure were excluded.

All patients underwent transvenous implantation of an atrial lead in the right atrium, high voltage right ventricular (RV) lead in the RV apex, and LV lead in a postero-lateral or lateral tributary of the coronary sinus. Septal and apical LV stimulation were avoided when feasible. The CRT therapy began immediately after the implant. Permanent CRT programming of AV and VV delays and LV stimulation vector was optimized individually for each patient as per the discretion of treating electrophysiologist based on the intrinsic PR interval and paced QRS morphology. In addition, VA detection and therapy programming were all standardized depending on the primary or secondary prevention ICD indication. All patients provided written informed consent and the study was approved by the research ethics board at University Health Network, Toronto.

### ECG recording and pacing protocol

The TWA measurements were made within the first 3 months after CRT therapy initiation (baseline) and then at least 6 months after the initial study (follow-up). The peri-implant period (until 1 month after implant) was avoided for the baseline study to minimize any spurious results from changing autonomic tone arising from volume shifts, fasting, analgesia, incisional pain, or stress related to the procedure. After careful skin preparation, standard 12-lead ECG electrodes were applied and continuous ECG was recorded in the recumbent position using a digital 12-lead Holter monitor (CardioMem CM 3000-12BT, Getemed Inc., Teltow, Germany) at 1024Hz sample rate (0.05–120Hz analogue bandwidth, ±6 mV voltage range, 12-bit digital resolution, 2.9μV least significant bit). Atrial (AAI) pacing serially at 100, 110 and 120 bpm for 3-minute duration at each rate, followed by atrio-biventricular (DDD-CRT) pacing in similar rate sequence, was performed through non-invasive wireless telemetry. A recovery period of 5-minutes of DDD-CRT pacing at the intrinsic heart rate was set in-between the AAI and DDD-CRT pacing sequels to minimize any confounding effects from rapid pacing induced APD alternans hysteresis [6].

To ensure comparable research DDD-CRT pacing with permanent CRT programming, the AV and VV delays and optimal LV stimulation vector selected for permanent CRT pacing were employed at both baseline and follow-up pacing studies.

### T-wave alternans analysis

Microvolt TWA was measured from the surface ECG using the spectral method and custom interactive software written in Matlab (MathWorks, Inc., Natick, Massachusetts) [7,8]. The details of our spectral method and its prior validation with the clinical tool (HeartWave system, Cambridge Heart Inc.) in 9 additional patients are provided in the S1 File. The ECG series was analyzed for T-wave amplitude alternans in segments of 128-beats, starting from the first beat and then moving in 128-beat segments sequentially by 16 beats over the 3-minute period. The presence of significant alternans in an ECG segment was determined on the basis of any alternans magnitude (V_alt_>0 μV) exceeding the mean noise level by >3 standard deviation (SD). The absolute V_alt_ value was not considered in the definition of significant alternans because T wave amplitude and APD gradients are different during ventricular pacing and native rhythm, and unlike the V_alt_ (≥1.9 μV) in the native recording, there is no clinically validated threshold of V_alt_ during DDD-CRT pacing that identifies a patient population at high risk of clinical events [8]. Accordingly, TWA magnitude (V_alt_ > 0 μV with k≥3), its duration (d), and burden (V_alt_·d, calculated as the area under curve of V_alt_ versus d) were quantified segment-wise in each 128-beat segment for all 12-ECG leads. The number of segments with positive TWA (V_alt_>0 μV, k ≥3) was indexed as a percentage of the total number of analyzed segments per 3-minute recording. The peak TWA magnitude, longest duration and peak burden across all 12 ECG channels were determined. For the purpose of the study, TWA during AAI pacing was considered as native TWA. Clinically significant native TWA was defined as V_alt_ ≥1.9 μV with k ≥3, in ≥2 contiguous ECG leads for the same segment at pacing rate of ≤110 bpm [8].

### Patient follow-up

All patients received guideline recommended heart-failure treatment and were followed prospectively. Reprogramming of AV or VV-delay and LV stimulation vector between the baseline and follow-up study was permitted if indicated clinically. All medications including anti-arrhythmics were continued. Patients were monitored at regular 3 to 6 monthly intervals post-CRT implant for clinical response and VA events. Clinical response to CRT was defined as an increase in 2D echocardiographically-derived volumetric LVEF by ≥5% leading to a final LVEF of ≥20% at ≥6-months post-CRT [9]. The echocardiogram readers were blinded to clinical outcomes. VA event was defined as the occurrence of sustained VA (>30 seconds), or appropriate ICD therapies.

### Statistical methods

Continuous patient variables were reported as mean ± SD or median (interquartile range [IQR]) and categorical variables were expressed as counts or percentages. To account for repeated measurements at 3 different pacing rates for each pacing type (AAI or DDD-CRT), changes in TWA measurements between the pacing types (AAI_Baseline_ vs. DDD-CRT_Baseline_, AAI_Baseline_ vs. DDD-CRT_Follow-up_, AAI_Baseline_ vs. AAI_Follow-up_ and DDD-CRT_Baseline_ vs. DDD-CRT_Follow-up_) were evaluated using the mixed effects models. In each model, pacing rate, pacing type and study time were included as repeated measure as appropriate, with patient treated as the random effect and pacing type as the fixed effect. Estimates of TWA measurements were expressed as mean ± standard error of mean (SEM), and their differences between the pacing types were determined. In addition, the dependency of these differences on the pacing rate was evaluated using an interaction term pacing type*pacing rate or study time*pacing rate, as appropriate, as a fixed effect in separate models. Furthermore, any interaction between the differences in pacing type with clinical response was tested in independent models using an interaction term pacing type*clinical response or study time*clinical response, as appropriate. Interaction with VA events was similarly tested. A two-tailed p-value of <0.05 was considered statistically significant, while a p-value of <0.1 was considered indicative of a statistically significant interaction. All calculations were performed using SPSS statistical software version 23 (IBM Corp., NY).

## Results

### Patient and CRT characteristics

Clinical characteristics and CRT programming of patients at the time of CRT implant are presented in Table 1 and Table 2, respectively. Twenty-four patients had received a quadripolar LV lead while the remaining had a bipolar LV lead.

**Table 1:** Patient characteristics.

Characteristic		Total (N = 40)	Responders (N = 27)*	Non-responders (N = 11)*	p value
**Age (years)**		65±11	65±11	62±10	0.4
**Male, n (%)**		25 (63)	17 (63)	8 (73)	0.6
**Cardiomyopathy, n (%)**	**Non-ischemic**	27 (68)	17 (63)	8 (73)	0.6
	**Ischemic**	13 (32)	10 (37)	3 (27)	
**Left ventricular ejection fraction (%)**		24±7	24±6	23±11	0.9
**NYHA Class**	**I**	3 (8)	3 (11)	0 (0)	0.7
	**II**	20 (50)	14 (52)	7 (64)	
	**III**	17 (42)	10 (37)	4 (36)	
**Rhythm**	**Sinus**	37 (92)	24 (89)	9 (82)	0.8
	**Atrial fibrillation**	3 (8)	2 (7)	1 (9)	
**Native QRS duration, ms**		166±31	166±31	162±33	0.6
**Native QRS morphology, n (%)**	**LBBB**	36 (90)	24 (89)	11 (100)	0.5
	**RBBB/IVCD**	4 (10)	3 (11)	0 (0)	
**History of atrial fibrillation**		6 (15)	3 (11)	2 (18)	0.8
**History of sustained VA, n (%)**		3 (8)	2 (7)	1 (11)	1.0
**Medications n (%)**	**β blocker**	39 (98)	26 (96)	11 (100)	All p>0.5
	**ACE Inhibitor/ARB**	39 (98)	26 (96)	11 (100)	
	**Spironolactone**	30 (75)	20 (74)	8 (73)	
	**Digoxin**	7 (18)	5 (19)	2 (18)	
	**Amiodarone**	8 (20)	5 (19)	3 (27)	

* Comparison between responders and non-responders to CRT is based on the 38 patients who had a follow-up echocardiographic examination.

ACE = angiotensin converting enzyme, ARB = angiotensin receptor blocker, CRT = cardiac resynchronization therapy, IVCD = intraventricular conduction defect, LBBB = left bundle branch block, RBBB = right bundle branch block, VA = ventricular arrhythmia. Values in xx±xx form represent mean±SD

**Table 2:** Basic CRT parameters at implant.

Parameter		Total
**Left ventricular lead position, n (%)**	**Posterolateral**	29 (72)
	**Lateral**	10 (25)
	**Septal**	1 (3)
	**Basal**	10 (25)
	**Mid**	30 (75)
	**Apical**	0 (0)
**Left ventricular pacing configuration, n (%)**	**Bipolar**	21 (53)
	**Extended bipolar (LV to RV coil)**	19 (47)
**Sensed AV delay, ms**		156±23
**Paced AV delay, ms**		116±18
**VV delay, ms**		32±16
**Paced QRS duration, ms**		152±36

Values in xx±xx form represent mean±SD

Patients were evaluated at a median of 2 months (IQR 1 to 2) (baseline TWA study) and 8 months (IQR 7 to 12) (follow-up TWA study) post-CRT implant. Of the 8 patients on amiodarone at baseline, 2 were taken off and 1 additional patient started amiodarone before the follow-up study. Six patients underwent clinically indicated reprogramming of AV/VV delays and 1 patient was programmed to VVT mode due to persistent atrial fibrillation between the baseline and follow-up study. No patient required repositioning of LV lead. Patients received an average of 94±14% and 96±14% biventricular pacing before the baseline and follow-up study respectively.

### Changes in QRS with CRT

The QRS duration reduced from 166±31ms (unpaced) to 152±36ms (CRT paced) at the time of implant (p = 0.003), with 24 (60%) patients achieving a dominant R-wave in lead V1 and S-wave in leads I and V6. The native QRS duration at follow-up was 164±21 ms, which was similar to the baseline unpaced QRS duration (p = 0.2).

### Baseline TWA study

Of the 40 patients, AAI pacing could not be performed in 13 patients (32%) at the baseline study due to atrial fibrillation (n = 3) or AV block during atrial pacing ≥ 100/min (n = 10). In the remaining patients, who satisfactorily completed at least one AAI pacing protocol, native TWA was clinically significant in 65% of subjects. In these patients, TWA was present in 70±7% of recorded 128-beat segments with a peak magnitude of 8±1 μV, longest duration of 143±7 seconds and peak burden of 400±97 μV.sec.

**Fig 1:**
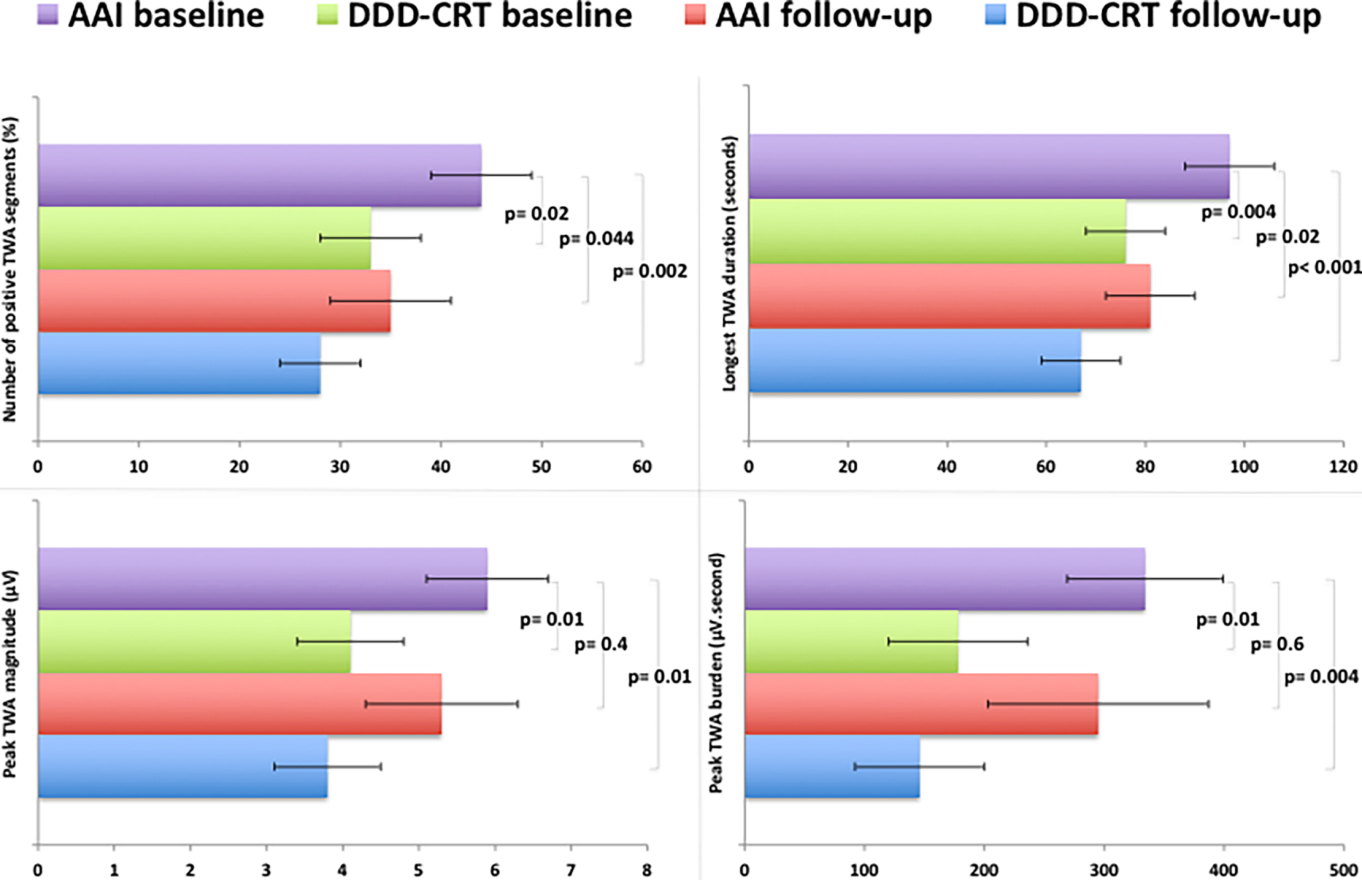
Change in measures of AAI (native) and DDD-CRT paced T- wave alternans (TWA) between baseline and follow-up studies. Each horizontal column peak and error bar along x-axis represents collective mean±SEM of all pacing rates.

Compared to native TWA at the baseline study, initiation of DDD-CRT instantly reduced the number of positive TWA segments by 10±4% (p = 0.02), peak TWA magnitude by 1.8±0.7 μV (p = 0.01), longest TWA duration by 20±7 seconds (p = 0.004) and peak TWA burden by 156± 60 μV.second (p = 0.01) (Table 3 and Fig 1). When comparing TWA at individual pacing rates, the reduction with DDD-CRT pacing was predominantly observed at 100 and 110 bpm but not at 120bpm (interaction p = 0.043 for number of positive TWA segments, 0.045 for peak magnitude, 0.009 for longest duration, and 0.045 for peak burden).

**Table 3:** Measures of TWA at baseline and follow-up.

Study		Baseline		Follow-up	
Parameter	Pacing Rate (bpm)	AAI	DDD-CRT	AAI	DDD-CRT
**Number of positive TWA segments (%)**	Collective	44±5	33±5*	35±6*	28±4*
	100	44±7	28±6^†^	30±7^†^	31±6
	110	44±7	30±6^†^	32±7^†^	24±6^†^
	120	42±7	43±6	46±7	27±6^‡^
**Peak TWA magnitude (μV)**	Collective	5.9±0.8	4.1±0.7*	5.3±1	3.8±0.7*
	100	4.8±1	3.4±0.9^†^	4.7±1.2	3.8±0.9
	110	6.9±1	3.8±0.9^†^	5.4±1.2	3.3±1^†^
	120	5.9±1	5.1±0.9	5.9±1.2	4.2±1
**Longest TWA duration (seconds)**	Collective	97±9	76±8*	81±9*	67±8*
	100	101±11	65±10^†^	74±11^†^	73±10
	110	99±11	73±10^†^	69±12^†^	62±10^†^
	120	89±12	91±10	105±12	66±10^‡^
**Peak TWA burden (μV.second)**	Collective	334±65	178±58*	295±92	146±54*
	100	224±88	131±78^†^	223±103	172±75
	110	386±90	152±78^†^	279±106	116±77^†^
	120	395±93	251±79	383±109	147±77^†^^‡^

* p<0.05 vs. baseline AAI study

† Differential effect of pacing rate in each comparison versus baseline AAI study (interaction p<0.1)

‡ Differential effect of pacing rate in comparison between baseline and follow-up DDD-CRT study (interaction p<0.1).

Refer to text for details.

Values represent mean±SEM

### Follow-up TWA study

Of the 40 patients, 12 patients (30%) could not complete AAI pacing at the follow-up study due to atrial fibrillation (n = 3), pacing induced atrial flutter (n = 1) or AV block during atrial pacing ≥ 100/min (n = 8). Of the 10 patients who had AV block at their baseline study, 6 patients exhibited AV block during follow-up study as well. In addition, 2 patients showed AV block for the first time at their follow-up study. The prevalence of clinically significant native TWA decreased from 65% at baseline to 52% at follow-up, but this difference did not reach statistical significance (p = 0.09). Additionally, compared to native TWA at baseline, the number of positive segments and longest-duration of native TWA diminished by 8±4% (p = 0.044) and 17±7sec (p = 0.02), respectively, during follow-up, with primary reduction observed at 100 and 110 bpm but not at 120bpm (interaction p = 0.047 for number of positive TWA segments, and 0.007 for longest TWA duration). The peak magnitude and peak burden of native TWA, however, remained unchanged (p = 0.4 and 0.6 respectively), at all pacing rates (interaction p = 0.4 and 0.2 respectively).

Progressive and greater reduction in all TWA measures was observed during DDD-CRT pacing at follow-up. Compared to native TWA at baseline, the number of positive TWA segments reduced by 14±4% (p = 0.002), peak TWA magnitude by 1.9±0.8 μV (p = 0.01), longest TWA duration by 27±8 seconds (p<0.001) and peak TWA burden by 176± 61 μV.second (p = 0.004) during DDD-CRT pacing (Table 3 and Fig 1). In addition, when comparing TWA at individual pacing rates, reduction with DDD-CRT pacing was observed at all pacing rates, with a predominant reduction at higher rates of 110 or 120bpm, than 100bpm (interaction p = 0.045 for number of positive TWA segments, 0.10 for peak magnitude, 0.017 for longest duration, and 0.037 for peak burden).

**Fig 2:**
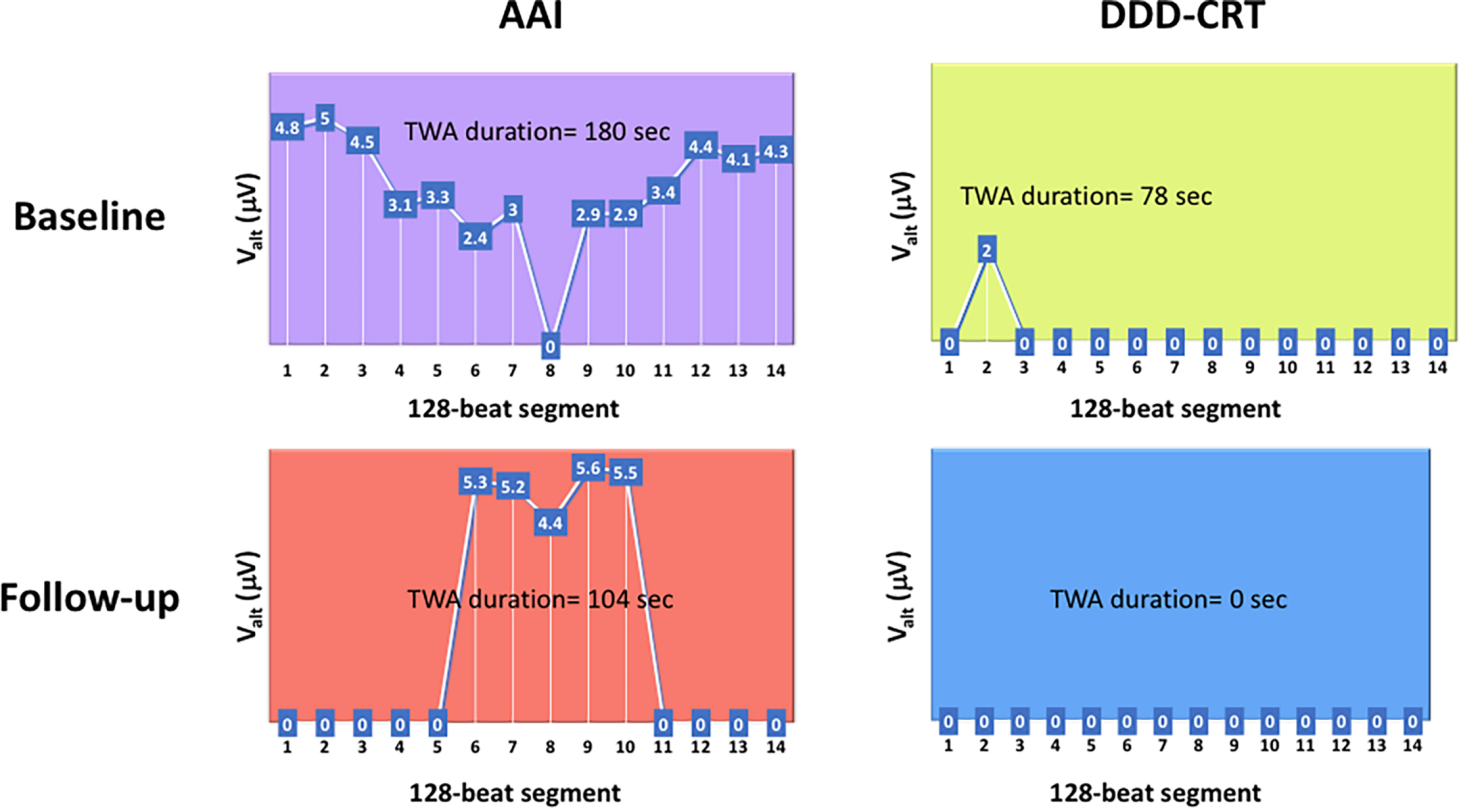
A case illustrating change in the number and duration of positive T-wave alternans (TWA) segments and their segment-wise TWA magnitude (V_alt_, k≥3) with long term CRT. Only TWA at 110 bpm in the ECG lead exhibiting longest duration of TWA is displayed for each pacing study. In each panel, x-axis represents sequence of 128-beat segments over the 3-minute duration, and y-axis represents TWA magnitude in μV. At baseline, compared to AAI-paced TWA, DDD-CRT pacing reduced instantaneously the number and duration of positive TWA segments as well as TWA magnitude. At follow-up, compared to AAI-paced TWA at baseline, the number and duration of positive TWA segments during AAI pacing reduce, however TWA magnitude remains unchanged. DDD-CRT pacing at follow-up shows complete absence of TWA.

Moreover, there was a trend towards reduction in the number of positive TWA segments between baseline and follow-up DDD-CRT pacing (p = 0.096). Notably, a significant reduction in various TWA measures was observed between baseline and follow-up DDD-CRT pacing at a higher pacing rate of 120 bpm (interaction p = 0.03 for number of positive TWA segments, 0.10 for longest duration, and 0.10 for peak burden). A representative case example of the TWA measures at baseline and follow-up with AAI and DDD-CRT pacing is shown in Fig 2.

### Relationship of TWA with clinical improvement after CRT

Echocardiographic reassessment of LVEF was done in 38 of 40 (95%) patients at median 10 months (IQR 7 to 15) after CRT implant. In these patients, LVEF improved from 24±7% pre-implant to 34±10% (p<0.0001) at follow up. The LVEF recovered by ≥5% leading to a final LVEF of ≥20% in 27 of 38 (71%) patients who were regarded as CRT responders. Two patients who had medical assessments without echocardiogram were excluded from CRT response analysis.

There was no difference in the native QRS duration, LBBB prevalence and other baseline characteristics between responders and nonresponders to CRT (all p values ≥0.4) (Table 1). However, acute reduction in QRS duration with initiation of DDD-CRT pacing was slightly greater in responders than nonresponders [167±6ms to 153±7ms (8% reduction) vs. 162±10ms to 151±12ms (7% reduction), interaction p = 0.03]. There was no difference in the change in native QRS duration at follow-up compared to the baseline unpaced QRS duration between responders and nonresponders (interaction p = 0.2).

**Fig 3:**
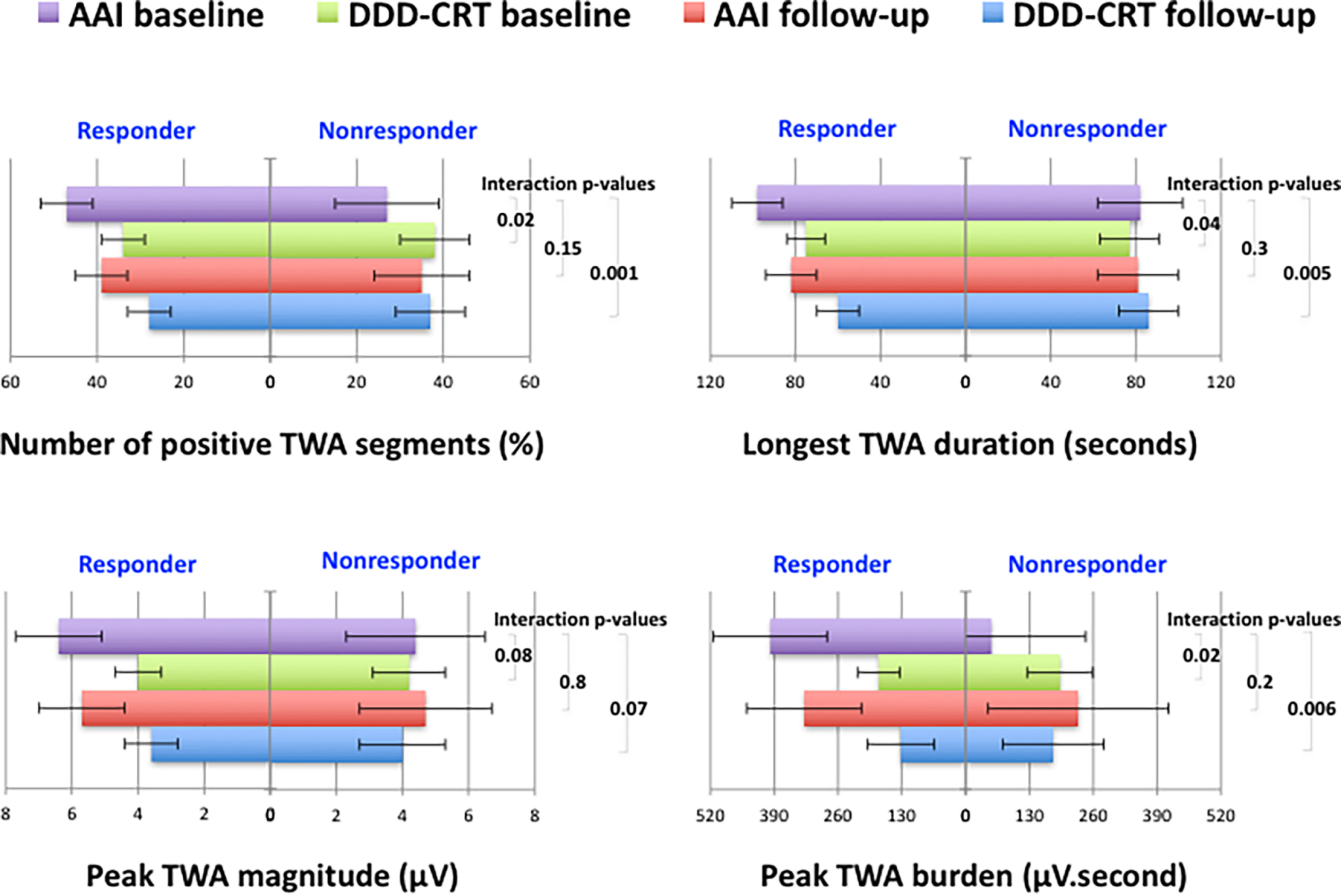
Change in measures of AAI (native) and DDD-CRT paced T- wave alternans (TWA) between baseline and follow-up studies in responders and nonresponders to CRT. Each horizontal column peak and error bar along x-axis represents collective mean±SEM of all pacing rates. P-values represent differential effect of CRT-response on comparison of TWA measures between different pacing types.

Fig 3 shows the comparison of various TWA measures between CRT responders and non-responders at baseline and follow-up. Compared to native TWA at the baseline study, reduction in TWA measures with initiation of DDD-CRT at baseline study was observed only in patients who responded to CRT, whereas TWA remained the same or became worse in nonresponders (all interaction p-values < 0.1). In addition, compared to native TWA at baseline, reduction in DDD-CRT paced TWA measures at follow-up was seen only in responders whereas it remained the same or became worse in nonresponders (all interaction p-values < 0.1). However, there was no statistically significant interaction between clinical response and change in native TWA measures between baseline and follow-up (all interaction p-values > 0.1), although peak native TWA burden at follow-up tended to be numerically worse than that at baseline among nonresponders.

### Relationship of TWA with VA events

After a median follow-up of 15 months (IQR 8 to 25), 3 (7.5%) patients received appropriate ICD therapy for VA, and 1 patient died of cancer. Patients with and without VA events had similar reduction in TWA at baseline and long-term CRT (all interaction p ≥ 0.2).

## Discussion

Our study demonstrated that immediate and long-term electric resynchronization with CRT reduces microvolt TWA in cardiomyopathy patients with intrinsic conduction disease and heart-failure. The main findings of the study are: 1) Initiation of CRT immediately reduces the prevalence and magnitude of TWA. 2) Progressive reduction in prevalence and magnitude of DDD-CRT paced TWA is observed after long-term CRT. 3) Long-term CRT also reduces the prevalence of native TWA but its magnitude does not change. 4) Reduction in DDD-CRT paced TWA is strongly related to clinical response to CRT.

### Electrical remodeling after CRT

#### Immediate effects

Electrical resynchronization of the ventricles with CRT is mediated by fusion of RV and LV propagating wavefronts. As a result, any pre-existing lines of functional conduction block disappear which reduces global activation time mainly in the LV epicardium [10]. Notably CRT paced QRS narrows by <20% compared to unpaced QRS in ~60% of patients [11,12]. In our study, initiation of CRT pacing acutely reduced QRS duration by 8% in responders and 7% in nonresponders.

CRT however, does not alleviate prolonged endocardial conduction time in the LV, and epicardial to endocardial dysynchronous activation may persist that can enhance transmural dispersion in tissue refractoriness [10]. Initiation of CRT therefore can produce adverse prolongation of QT and T_peak_-T_end_ intervals [13]. Despite these limiting factors, initiation of CRT in our cardiomyopathy population immediately diminished both magnitude and prevalence of pacing induced TWA during the baseline study, specifically in patients who eventually showed CRT response. A plausible mechanism for this finding may involve conduction velocity restitution, which describes the relationship of conduction velocity to the coupling interval of an extrastimulus or the rate of steady state pacing. With rapid pacing, conduction velocity will decrease; thereby increasing activation time and the diastolic interval at sites downstream of pacing. Because APD is dependent on the preceding diastolic interval, the phase of repolarization alternans can be altered based on the tissue’s conduction velocity restitution properties [14]. In experimental and simulation studies, steep conduction velocity restitution function can augment cardiac alternans in the downstream tissues and promote spatially discordant alternans [15]. With acute CRT pacing as opposed to atrial pacing at the same rate, the effect of steep conduction velocity restitution to potentially augment repolarization alternans may be mitigated because LV pacing effectively reduces LV activation time and the diastolic interval of downstream LV tissue.

Immediate reduction in DDD-CRT paced TWA was seen specifically in patients who eventually had clinical response to CRT. A potential reason could be the preferential contribution of ventricular ion-current remodeling versus myocardial scarring to the cardiomyopathic substrate in these patients. Although we did not evaluate presence and amount of myocardial scar, it is conceivable that conduction delay in patients who exhibited immediate reduction in TWA was primarily due to ventricular electrical conduction remodeling that later responded clinically to CRT as well, thereby permitting immediate TWA reduction to predict mechanical LV function improvement. In contrast, failure to achieve immediate TWA reduction was possibly due to greater ventricular scar burden that may have also generated non-response to CRT and worse LVEF outcomes [16,17].

#### Long-term effects

Long-term CRT attempts to normalize the pattern of LV activation that ultimately can promote several structural and molecular changes in the cardiac electric substrate (i.e. mechano-electric feedback).[18] Besides restoring cardiomyocyte size, there is improvement in kinetics of intracellular calcium cycling through up regulation of SERCA, RyR, and sodium-calcium exchanger levels in patients responding to CRT while no significant change is observed in nonresponders [1,2]. Furthermore, Aiba et al demonstrated in an animal model of LV dyssynchrony, a reduction in inward rectifier potassium current and delayed rectifier potassium current, which was associated with APD prolongation [19]. These effects were partially restored after 3 weeks of CRT pacing, including shortening of APD.

At the 6-months follow-up study, we observed further reduction in the magnitude and prevalence of DDD-CRT paced TWA compared to the baseline native TWA. Moreover, the prevalence of native TWA also decreased significantly. These late changes in TWA predominated in CRT responders, while nonresponders not only failed to achieve equivalent reduction but also had worsening in both DDD-CRT paced and native TWA. There are several potential mechanisms that may explain these findings. Foremost, long-term CRT pacing in clinical studies up regulates SERCA, RyR, and sodium-calcium exchanger, which can improve intracellular calcium cycling and reduce cytosolic calcium alternans. Cytosolic calcium in turn can modulate APD via L-type calcium currents and the sodium-calcium exchanger. In experimental preparations, a reduction in cytosolic calcium alternans has been shown to reduce repolarization alternans. [1,2,16]

In addition, long-term CRT pacing can remodel several sarcolemmal ion channels, resulting in APD shortening and flatten of the APD restitution relationship [19]. In experimental studies, an APD restitution slope >1 promotes repolarization alternans, while a slope <1 suppresses repolarization alternans [16]. Accordingly, it is conceivable that a reduction of APD restitution slope may attenuate TWA after long-term CRT.

Another plausible mechanism for long-term CRT induced-TWA remodeling involves weakening of LBBB related long-term cardiac memory, which can also modulate repolarization alternans. In heart failure patients with LBBB, the abnormal vector of activation can create large spatial dispersions in APD between early and late-activated segments. By altering the direction of activation with long-term CRT, these spatial APD gradients can also resolve as is evident by a change in T wave amplitude and morphology [3,4,18,20,21]. Resolution of APD gradients can suppress repolarization alternans [16]. Accordingly, we speculate that the progressive reduction in native TWA observed more than 6-months after chronic CRT pacing may also be attributed in part to reversal of LBBB-related repolarization dispersion from long-term cardiac memory, that ultimately promoted homogenization of spatial APD gradients.

### Previous assessment of TWA in the CRT population

Ehrlich et al showed 83% concordance between microvolt TWA test results obtained during right atrial and DDD-CRT pacing at the time of CRT implant [22]. However, TWA was interpreted in a dichotomized manner as either positive or negative, despite the fact that there is no clinically validated threshold of V_alt_ during biventricular pacing that identifies a patient population at high risk of clinical events [8]. Immediate reduction in the prevalence and magnitude of TWA with CRT has been reported previously in cardiomyopathy patients by Anh et al [20]. In their study, peak TWA magnitude was assessed from a single vector lead at 6 weeks post CRT-implant. Native TWA magnitude during AAI pacing (2.0±1.9 μV) was small, and a slim but significant reduction was observed with biventricular pacing. Our results at baseline study were generally in accordance with these data. However, we observed a much larger peak magnitude of native TWA (5.9±0.8 μV) that reduced considerably by almost 2 μV with CRT pacing. Technical differences in ECG recording (12-lead vs. single lead), and more severe LV systolic dysfunction with wider QRS and thus advanced ventricular remodeling in our patient population may explain these differences compared to the previous study.

Recently, Hua et al reported 30% reduction in the prevalence of alternans in local epicardial LV electrogram-T wave during AAI pacing after 3 months of CRT [23]. Patients with and without reduction in TWA appeared to derive similar improvement in LVEF, suggesting no relationship between heart-failure improvement and CRT-reduced TWA. In contrast, our study reports detailed immediate and longer-term changes in both prevalence and magnitude of native and CRT-paced TWA recorded from the surface 12-lead ECG, and beneficial TWA remodeling particularly in CRT functional responders.

### Implications for CRT therapy

We demonstrated that CRT pacing reduced TWA impressively in cardiomyopathy patients, primarily in those who had mechanical LV function improvement with CRT. Importantly, reduction in TWA in CRT responders was observed immediately at the baseline study well before LVEF recovery may have occurred. This beneficial TWA response was sustained and progressive as confirmed at the follow-up TWA study. Such reductions in TWA were seen despite minimal reduction in CRT-paced and native QRS duration. Immediate and long-term change in TWA particularly with DDD-CRT pacing can therefore be an important prognostic marker for mechanical recovery after CRT.

#### TWA and risk of VA in CRT population

Treatment of LV conduction delay with CRT does not counter long trans-septal conduction time, and LV endocardial dysynchrony may persist [10]. Moreover, by reversing the normal endocardial to epicardial sequence of depolarization, LV epicardial stimulation may promote transmural dispersion of repolarization. This can enhance the development of VA, which has been observed in heart failure patients with prior VA and non-response to CRT [13,24]. However, there is significant reduction in the risk of sudden death among responders to CRT in large studies [24]. Although VA risk was not influenced by the reduction in TWA in our study patients, the clinical translation of TWA remodeling with CRT on reducing VA burden merits further study in a larger cohort. In other small studies of cardiomyopathy patients with a native wide QRS, however, neither AAI-paced nor CRT-paced TWA were found to be useful for risk stratification despite equally prevalent TWA compared to patients with narrow QRS [20,25].

### Limitations

The small sample size is a limitation, however significant differences between baseline and follow-up TWA study were demonstrable. Fewer VA events in our cardiomyopathy cohort precluded any meaningful analysis of clinical benefit of long-term CRT-induced TWA remodeling on VA burden. Second, baseline study performed at a median 2 months after CRT initiation may not have captured true baseline TWA as some patients may already have had substantial electrical and mechanical remodelling within weeks of CRT [18,26–29]. However, Cvijic et al demonstrated minimal early structural remodeling such that LV end systolic volume did not change before 3 months of CRT [30]. Despite this, a further decrease in TWA at the follow-up study supported our primary hypothesis that long-term CRT attenuates TWA in cardiomyopathy patients. Third, we could not discern whether late reduction in native TWA was principally due to reverse cardiac remodeling through CRT or co-interventions such as medical therapy for heart failure, in particular beta-blockers [31]. However, cardiac medications were titrated fairly uniformly across patients post CRT according to heart failure management guidelines [32]. Finally, acute reduction in T wave amplitude with CRT pacing compared to AAI pacing would reduce TWA signal-to-noise ratio and may have accounted for the attenuation of TWA observed at the baseline study [20]. However, progressive reduction in both native and DDD-CRT TWA during follow-up compared to native TWA at baseline argues against T wave signal resolution as the primary cause of diminishing TWA with CRT.

## Conclusions

Long-term CRT reduces the prevalence of native TWA without changing native QRS duration. Sustained reduction in the prevalence and magnitude of DDD-CRT paced TWA is also observed. These findings provide evidence for long-term CRT induced repolarization remodeling in patients with cardiomyopathy. The effect is closely dependent on CRT response and is a marker of mechanical recovery of LV with CRT.

## Supporting information

S1 File Supplemental methods. (DOCX)
