# Supplementary material for: Reduced T wave alternans in heart failure responders to cardiac resynchronization therapy: Evidence of electrical remodeling
Source: PLoS One. 2018 Jun 28;13(6):e0199637. doi: 10.1371/journal.pone.0199637 (PMC6023131; doi:10.1371/journal.pone.0199637)
Supplement: S1 File — (DOCX) [file pone.0199637.s001.docx]

**Data Supplement**

**Reduced T wave alternans in heart failure responders to cardiac resynchronization therapy: Evidence of electrical remodeling**

**Supplemental methods**

**Spectral method for T wave alternans analysis**

A template low noise QRST complex of each pacing type (AAI or DDD-CRT) was manually defined and compared to each QRST complex in the respective 3-minute recording. To eliminate ectopics, fusion beats and loss of capture, complexes that were <90% similar to the morphology of the template complex were excluded from analysis. To eliminate noisy beats, QRST complexes with an ST segment root mean square noise level >10µV were excluded from analysis. The baseline wander was removed by subtracting an interpolated cubic spline. Each eliminated beat was replaced with an averaged QRST complex. Contiguous beats were aligned to their QRS complex, maximizing the dot product to the averaged QRS complex. The restructured ECG series were analyzed for T-wave amplitude alternans in segments of 128-beats, starting from first beat and then moving in 128-beat segments sequentially by 16 beats over the 3-minute period. For each segment, a 2-dimensional matrix was constructed with 128 rows corresponding to the 128 beats and T-wave amplitude in *n* columns, where *n* represented the number of sampled time points in the JT interval. Fast Fourier Transform was applied to the amplitude series column-wise to generate power spectra for each time point, which were then summed to generate an aggregate power spectrum. The spectral magnitude at a frequency of 0.5 cycles/beat represents the alternans magnitude (P_0.5_). The noise band was defined as the 10 preceding spectral points (0.33 to 0.48 cycles/beat). The mean amplitude in the noise band was the mean noise (μ_noise_) and the standard deviation (SD) of noise was σ_noise_. The magnitude of alternans was measured by V_alt_ and k value:

V_alt_ = √ (P_0.5_ - μ_noise_) / JT duration

k = (P_0.5_ - μ_noise_) / σ_noise_

The presence of significant alternans in an ECG segment was determined on the basis of a k value of ≥3, indicating any alternans magnitude (V_alt_>0 μV) exceeding the mean noise level by more than 3 SDs. A sample ECG with V_alt_ of 11.1μV is shown in Supplemental Figure 1.

**Supplemental Figure 1.**

**
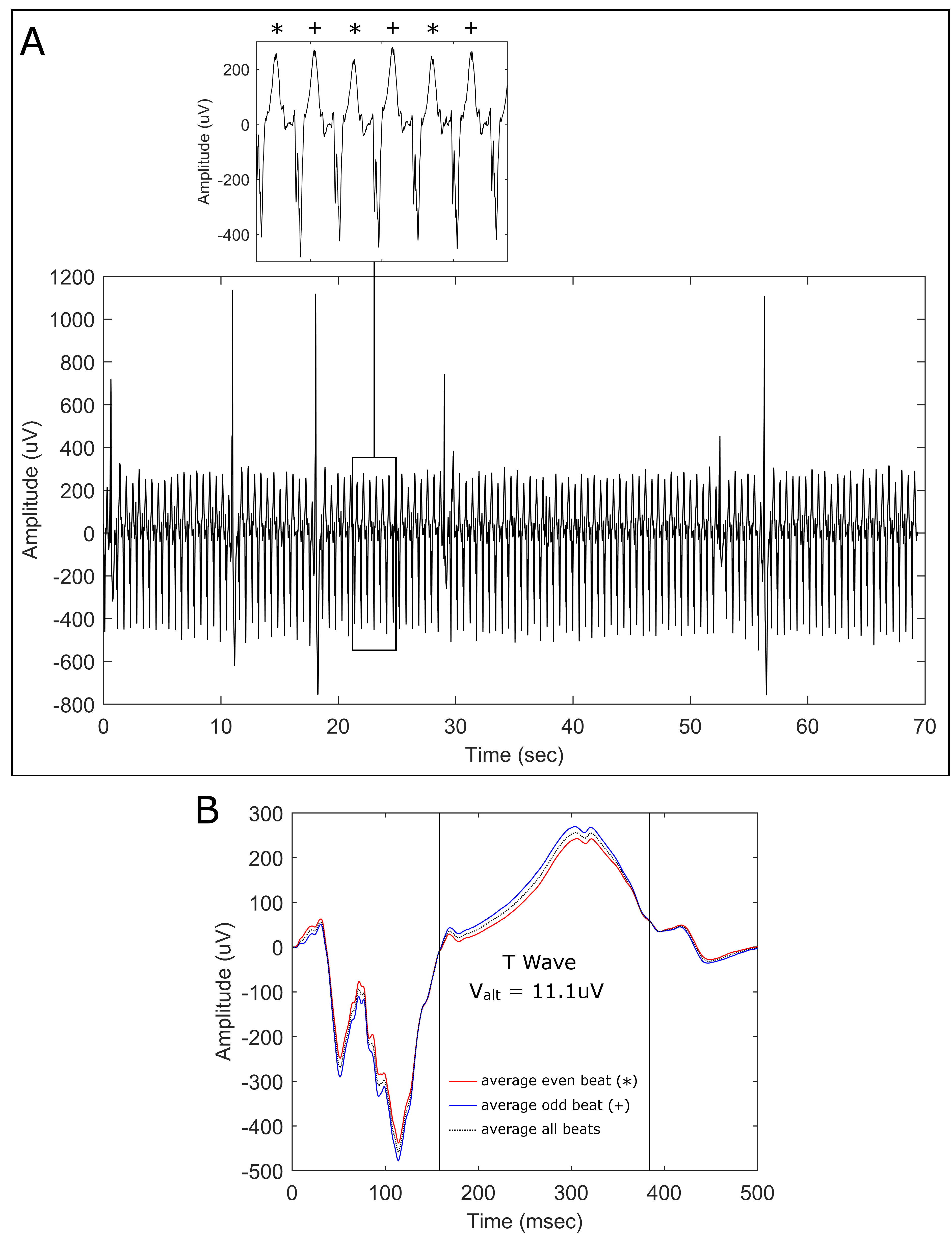
**

**Supplemental Figure 1. Illustrative example of TWA calculation from a representative patient**

(A) Illustration of a 128-beat segment from lead V5 of a representative patient. Using the spectral method, the TWA magnitude for this segment (V_alt_) was calculated as 11.1 μV. The magnified inset of 6 consecutive beats illustrates visible alternation in the T wave between even (*) and odd (+) beats.

(B) Illustration of the average of the even beats (red line), the odd beats (blue line) and all beats (black dashed line) in the 128-beat segment. Although the TWA V_alt_ is computed as 11.1 uV, the mean difference between the average odd and even beats is closer to double the V_alt_ at 21.9 uV. This is because V_alt_ is defined as the square root of the spectral power occurring at the alternans frequency (0.5 cycles/beat), which corresponds to the difference in voltage between the overall average beat and either the average even or average odd beat (i.e. half of the mean difference between the averaged even and odd beats).

**Validation of our TWA algorithm against HeartWave system**

Our TWA algorithm results were compared to those generated by the clinical tool, HeartWave™ (Cambridge Heart Inc.), amongst a separate cohort of 9 patients with cardiomyopathy who underwent TWA testing during electrophysiological study. Both methods are based on 12-lead T-wave amplitude spectral analysis of moving 128-beat segments and identical definitions of TWA signal to noise; however the clinical tool uses proprietary noise cancelling electrodes to improve TWA signal detection. The clinical tool identified 7 (78%) patients with clinically significant TWA compared to all 9 patients with our algorithm. The two patients without clinically significant had a V_alt_ > 0 µV but < 1.9 µV (k ≥3) at a heart rate of 100 bpm. Our algorithm found both of these patients to have a V_alt_ just above the 1.9 µV (k ≥3) threshold (2.1 µV and 3.7 µV respectively) at approximately the same times as the subthreshold detections by the clinical tool. The TWA_max_ obtained from the clinical tool was greater than the TWA_max_ calculated by our algorithm (7.1 ± 3.5 µV vs. 5.7 ± 2.6 µV, p=0.03), likely due to the use of proprietary noise cancelling electrodes. The onset heart rate for clinically significant TWA for all patients using either method was 100 bpm. Notably, there was good agreement in tracking of V_alt_ changes over time as demonstrated in Supplemental Figure 2.

**Supplemental figure 2**

**
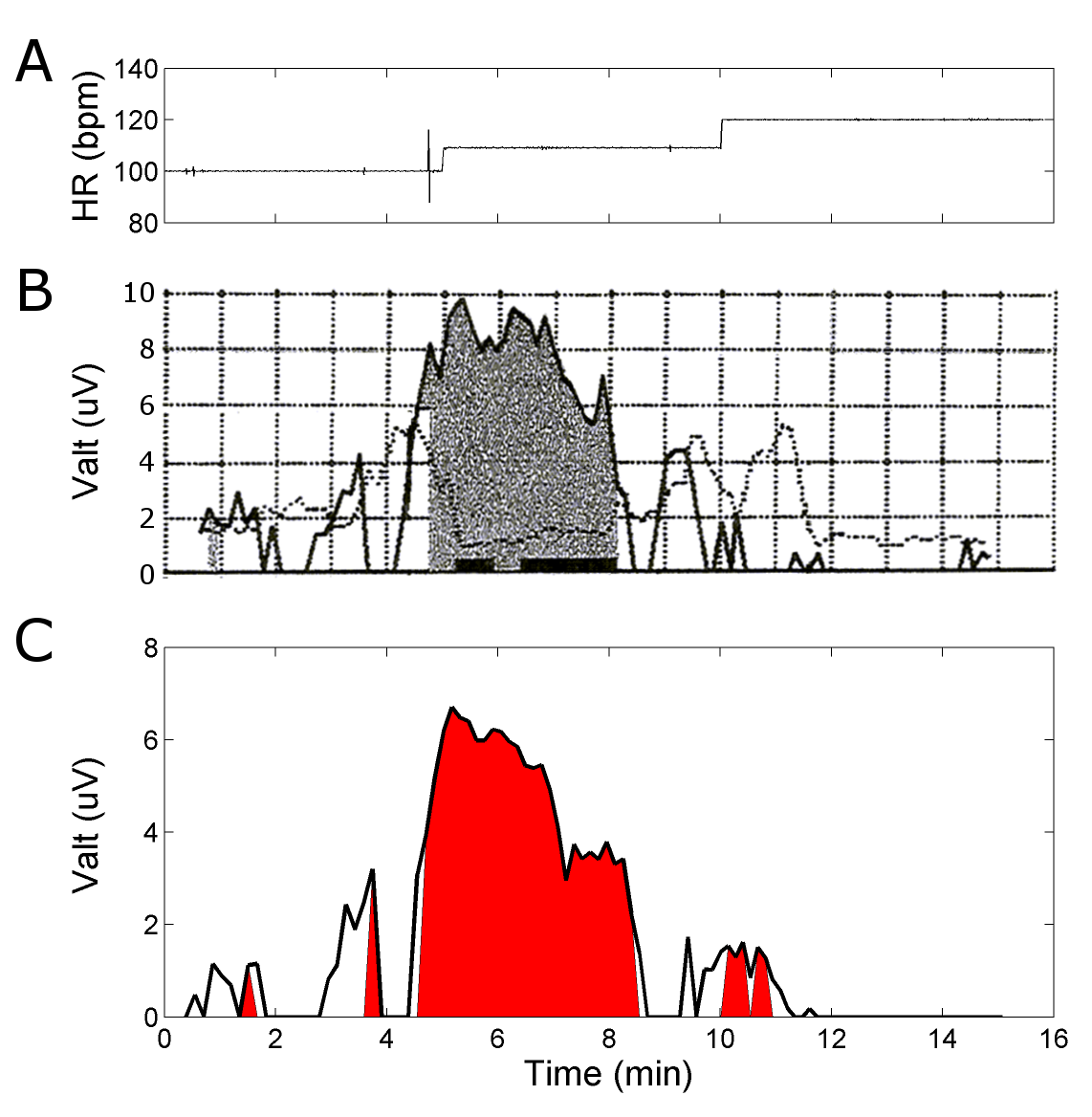
**

**Supplemental figure 2. Comparison of TWA detection between the clinical TWA analysis tool, the HeartWave™ (Cambridge Heart Inc.) and our algorithm in a representative patient.**

(A) Plot illustrating heart rate over the course of an entire TWA pacing protocol.

(B) Plot of TWA magnitude (V_alt_, solid black line) versus time in ECG lead V4 of a representative patient as outputted by the clinical tool. Grey shaded areas indicate TWA signal with k ≥3.

(C) TWA magnitude (V_alt_) plot created by our algorithm from ECG lead V4 of the same patient. Red shaded areas denote TWA signal with k ≥3.

In both V_alt_ graphs, transient TWA appear at 100 bpm but does not become sustained above the 1.9 µV threshold until the pacing rate is increased to 110 bpm. Alternans decreases and finally subsides when the pacing rate is increased to 120 bpm.
